# Comparative Proteomics of Outer Membrane Vesicles from Polymyxin-Susceptible and Extremely Drug-Resistant Klebsiella pneumoniae

**DOI:** 10.1128/msphere.00537-22

**Published:** 2023-01-09

**Authors:** Maytham Hussein, Raad Jasim, Hakan Gocol, Mark Baker, Varsha J. Thombare, James Ziogas, Aayush Purohit, Gauri G. Rao, Jian Li, Tony Velkov

**Affiliations:** a Monash Biomedicine Discovery Institute, Department of Microbiology, Monash University, Clayton, Victoria, Australia; b Department of Biochemistry and Pharmacology, School of Biomedical Sciences, Faculty of Medicine, Dentistry and Health Sciences, The University of Melbourne, Parkville, Victoria, Australia; c Discipline of Biological Sciences, Priority Research Centre in Reproductive Biology, Faculty of Science and IT, University of Newcastle, Callaghan, New South Wales, Australia; d Division of Pharmacotherapy and Experimental Therapeutics, Eshelman School of Pharmacy, University of North Carolina, Chapel Hill, North Carolina, USA; e Department of Pharmacology, College of Pharmacy, University of Babylon, Iraq; University of Rochester

**Keywords:** polymyxin B, OMVs, proteomics, MDR Gram-negative

## Abstract

Outer membrane vesicles (OMVs) secreted by Gram-negative bacteria serve as transporters for the delivery of cargo such as virulence and antibiotic resistance factors. OMVs play a key role in the defense against membrane-targeting antibiotics such as the polymyxin B. Herein, we conducted comparative proteomics of OMVs from paired Klebsiella pneumoniae ATCC 700721 polymyxin-susceptible (polymyxin B MIC = 0.5 mg/L) and an extremely resistant (polymyxin B MIC ≥128 mg/L), following exposure to 2 mg/L of polymyxin B. Comparative profiling of the OMV subproteome of each strain revealed proteins from multiple perturbed pathways, particularly in the polymyxin-susceptible strain, including outer membrane assembly (lipopolysaccharide, *O*-antigen, and peptidoglycan biosynthesis), cationic antimicrobial peptide resistance, β-lactam resistance, and quorum sensing. In the polymyxin-susceptible strain, polymyxin B treatment reduced the expression of OMV proteins in the pathways related to adhesion, virulence, and the cell envelope stress responses, whereas, in the polymyxin-resistant strain, the proteins involved in LPS biosynthesis, RNA degradation, and nucleotide excision repair were significantly overexpressed in response to polymyxin B treatment. Intriguingly, the key polymyxin resistance enzymes 4-amino-4-deoxy-l-arabinose transferase and the PhoPQ two-component protein kinase were significantly downregulated in the OMVs of the polymyxin-susceptible strain. Additionally, a significant reduction in class A β-lactamase proteins was observed following polymyxin B treatment in the OMVs of both strains, particularly the OMVs of the polymyxin-susceptible strain. These findings shed new light on the OMV subproteome of extremely polymyxin resistant K. pneumoniae, which putatively may serve as active decoys to make the outer membrane more impervious to polymyxin attack.

**IMPORTANCE** OMVs can help bacteria to fight antibiotics not only by spreading antibiotic resistance genes but also by acting as protective armor against antibiotics. By employing proteomics, we found that OMVs have a potential role in shielding K. pneumoniae and acting as decoys to polymyxin attack, through declining the export of proteins (e.g., 4-amino-4-deoxy-l-arabinose transferase) involved in polymyxin resistance. Furthermore, polymyxin B treatment of both strains leads to shedding of the OMVs with perturbed proteins involved in outer membrane remodeling (e.g., LPS biosynthesis) as well as pathogenic potential of K. pneumoniae (e.g., quorum sensing). The problematic extended spectrum beta-lactamases SHV and TEM were significantly reduced in both strains, suggesting that polymyxin B may act as a potentiator to sensitize the bacterium to β-lactam antibiotics. This study highlights the importance of OMVs as “molecular mules” for the intercellular transmission and delivery of resistance and cellular repair factors in the bacterial response to polymyxins.

## INTRODUCTION

Extremely drug-resistant (XDR) K. pneumoniae is a Gram-negative “superbug” responsible for numerous nosocomial and community outbreaks (1, 2). This pathogen often displays resistance toward β-lactam antibiotics (e.g., third-generation cephalosporins and carbapenems), through the production of novel extended-spectrum β-lactamases (ESBL), carbapenemases, and the New Delhi *metallo*-β-lactamase 1 (NDM-1) (1, 3). The upregulation of efflux pump transport proteins such as AcrAB and OqxAB is another tactic used by K. pneumoniae to extrude antibiotics from their intracellular environment, thereby preventing the accumulation of bactericidal concentrations (4). Furthermore, resistance in K. pneumoniae has arisen through the means of modifying enzymes (e.g., aminoglycoside-modifying enzymes) (5, 6).

The two clinically available polymyxins, namely, colistin and polymyxin B, have been increasingly used as the last-line therapy against problematic XDR K. pneumoniae strains (7). Surveillance reports of antimicrobial resistance indicate that 98.2% of K. pneumoniae clinical strains remain susceptible to polymyxin B and colistin (8, 9). Notwithstanding, XDR strains resistant to polymyxins have recently emerged, which underscores the need for further investigations of polymyxin resistance mechanisms in XDR K. pneumoniae (10 to 13). The primary antimicrobial killing action of polymyxins is mediated through a direct interaction with the lipid A component of lipopolysaccharide (LPS), followed by the insertion of the polymyxin molecule into the fatty acyl layer of the outer membrane (OM) (7). The integration of the polymyxin molecules into the OM results in disruption of the selective permeability barrier and leakage of intracellular contents, which culminates in bacterial death (14). Polymyxin resistance in K. pneumoniae primarily involves modification of lipid A with 4-amino-4-deoxy-l-arabinose or phosphoethanolamine (15, 16). The addition of 4-amino-4-deoxy-l-arabinose modifications to the lipid A phosphates is under the control of the two-component regulatory systems PhoPQ-PmrD-PmrAB that are activated in response to cationic antimicrobial peptides (CAMPs), low pH, low magnesium, and high iron (17). Our group has also reported that the underacylation of lipid A increases the polymyxin susceptibility of K. pneumoniae (18, 19). Outer membrane proteins (OMPs) have also been implicated in OM remodeling that is associated with increased polymyxin susceptibility in K. pneumoniae (20).

Gram-negative bacteria can shed components of their OM via outer membrane vesicles (OMVs), which are spherical bilayer structures approximately 20 to 200 nm in diameter (21). Structurally, these vesicles are made up of OM, periplasmic lipids, and proteins (22). OMVs carry bacterial components as cargo, including cytoplasmic and inner membrane proteins, genetic materials, virulence factors, metabolites, and signaling molecules (23, 24). It has been recently found that the OMVs can mediate horizontal gene transfer events and thereby, help promote the development of antibiotic resistance in Gram-negative bacteria, including K. pneumoniae (25). Therefore, OMVs play crucial roles in bacterial virulence, inflammation, host immune stimulation, and of particular interest, in defense and resistance mechanisms against antibiotics (26). OMVs protect bacteria from the effects of antibiotics through the use of antibiotic-degrading enzymes (e.g., β-lactamase and aminoglycosides-inactivating enzymes), or by capturing membrane-active antibiotics such as polymyxins (26 to 29). This is shown by the fact that incubation of susceptible strains with OMVs from polymyxin-resistant Gram-negative bacteria conferred a protective effect for susceptible bacteria against polymyxins (29). This highlights the need to understand the compositional differences in the subproteome of OMVs from polymyxin-susceptible and extremely resistant K. pneumoniae isolates. In the present study, we primarily aimed to perform a comparative analysis of the OMV subproteome of paired polymyxin-susceptible and XDR polymyxin-resistant K. pneumoniae isolates and identify key proteins that are selectively packaged from the parent bacteria into the OMV subproteome. The garnered data provide a novel understanding of the OMV subproteome associated with extreme levels of polymyxin B resistance in the problematic Gram-negative opportunistic pathogen, K. pneumoniae.

## RESULTS AND DISCUSSION

### OMV subproteome characterization.

**FIG 1:**
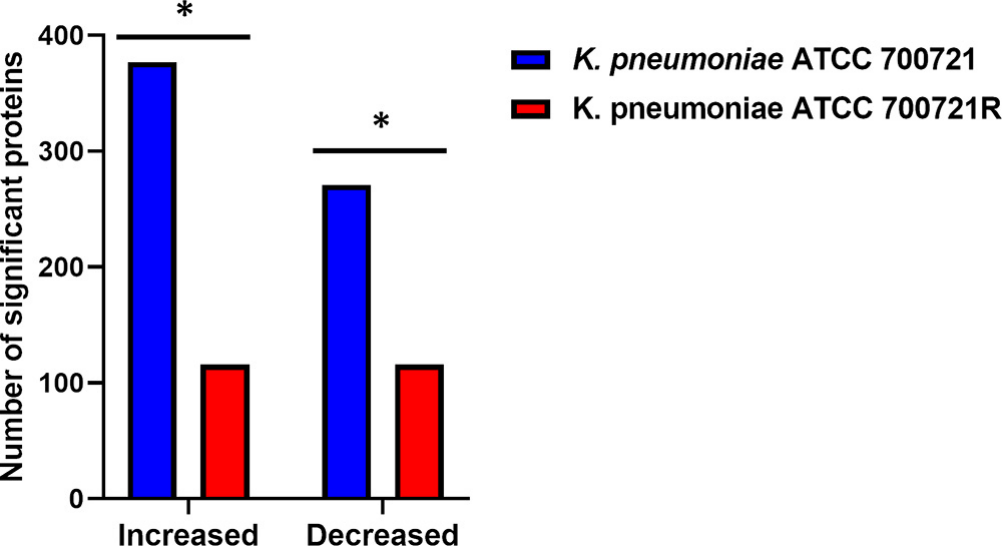
Number of significantly changed OMVs proteins from K. pneumoniae ATCC 700721 and K. pneumoniae ATCC 700721R after polymyxin B therapy (*n *= 3). *, >1.0-log_2_FC, *P ≤ *0.05; FDR ≤ 0.05.

Proteomic analysis of the OMVs identified 1,115 and 1,121 differentially represented proteins in the polymyxin-susceptible K. pneumoniae ATCC 700721 and its paired XDR strain, respectively. Subcellular localization and virulence prediction of the OMV subproteomes are explained in detail in the supplemental document (Text S1; Fig. S1 and S2). Comparative analysis (≥1-log_2-fold_ change [FC]; *P ≤ *0.05; FDR ≤ 0.05) displayed that a total of 648 proteins (271 downregulated and 377 upregulated proteins) were significantly changed in the OMVs of the susceptible strain, compared to 232 significantly changed proteins (116 downregulated and 116 upregulated proteins) in the OMVs from the resistant strain (Fig. 1). Polymyxin B treatment induced 15 and 16 uniquely perturbed OMVs proteins in the OMV subproteomes of polymyxin-susceptible K. pneumoniae ATCC 700721 and polymyxin-resistant K. pneumoniae ATCC 700721R, respectively (Table 1; Fig. S3). As elaborated below, the differentially expressed OMV proteins were involved in cell envelope biosynthesis (LPS, peptidoglycan, *O*-antigen), protein export systems, two-component systems (TCS), cationic antimicrobial peptide (CAMP) resistance proteins, β-lactam resistance, quorum sensing, RNA degradation, nucleotide excision/repair, and protein export.

**TABLE 1:** Unique OMVs proteins perturbed in polymyxin-susceptible K. pneumoniae ATCC 700721 and polymyxin-resistant K. pneumoniae ATCC 700721R following polymyxin B treatment

Protein ID	Gene	Protein	Subcellular localisation
K. pneumoniae ATCC 700721			
A6T5B0	proC	Pyrroline-5-carboxylate reductase	Cytoplasmic
A6T7E0	KPN78578	UPF0502 protein KPN78578_10500	Cytoplasmic
A6T7H8	ycfX	N-acetyl-d-glucosamine kinase	Cytoplasmic
A6T7T9	ydjQ	Endonuclease in nucleotide excision repair	Cytoplasmic
A6T7X7	yciL	Pseudouridine synthase	Cytoplasmic
A6T7G4	lpoB	Penicillin-binding protein activator LpoB (PBP activator LpoB)	Cytoplasmic membrane
A6T4X9	bamA	Outer membrane protein assembly factor BamA	Outer membrane
A6T5E4	tsx	Nucleoside-specific channel-forming protein Tsx	Outer membrane
A6T631	fepA	Outer membrane porin, receptor for ferric enterobactin and colicins B and D	Outer membrane
A6T751	ompA	Outer membrane protein A	Outer membrane
A6T4Y0	hlpA	Periplasmic molecular chaperone for outer membrane proteins	Periplasmic
A6T509	mltD	Lytic murein transglycosylase C, membrane-bound	Periplasmic
A6T5J7	ybaY	Glycoprotein/polysaccharide metabolism	Periplasmic
A6T5R7	KPN_00488	5-hydroxyisourate hydrolase (HIU hydrolase) (HIUHase) (EC 3.5.2.17)	Periplasmic
A6T639	fepB	Ferric enterobactin (Enterochelin) binding protein periplasmic component	Periplasmic
K. pneumoniae ATCC 700721R			
A6T8I9	paaJ	Acetyl-CoA acetyltransferase	Cytoplasmic
A6TCI7	rpoE	RNA polymerase sigma-70 factor	Cytoplasmic
A6TCL4	rplS	50S ribosomal protein L19	Cytoplasmic
A6TEY0	rpsL	30S ribosomal protein S12	Cytoplasmic
A6TF49	glgC	Glucose-1-phosphate adenylyltransferase	Cytoplasmic
A6TFM7	rpmG	50S ribosomal protein L33	Cytoplasmic
A6TFN2	slmA	Nucleoid occlusion factor SlmA	Cytoplasmic
A6TBW5	nuoK	NADH-quinone oxidoreductase subunit K	Cytoplasmic membrane
A6TGH3	wzz	ECA polysaccharide chain length modulation protein	Cytoplasmic membrane
A6TGI6	hemX	Uroporphyrinogen III methylase	Cytoplasmic membrane
A6THX1	mdoB	Phosphoglycerol transferase I	Cytoplasmic membrane
A6TIT5	traE	F pilus assembly protein	Cytoplasmic membrane
A6TBG4	asmA	Suppressor of ompF assembly mutants	Outer membrane
A6TAE1	lpp	Murein lipoprotein	Outer membrane
A6TA04	sodB	Superoxide dismutase	Periplasmic
A6THZ1	osmY	Hyperosmotically inducible periplasmic protein	Periplasmic

10.1128/msphere.00537-22.5 TEXT S1 Subcellular localization and virulence prediction of the OMV subproteomes. Download Text S1, DOCX file, 0.03 MB. Copyright © 2023 Hussein et al. 2023 Hussein et al. https://creativecommons.org/licenses/by/4.0/ This content is distributed under the terms of the Creative Commons Attribution 4.0 International license.

### Lipopolysaccharide biosynthesis and modification.

**FIG 2:**
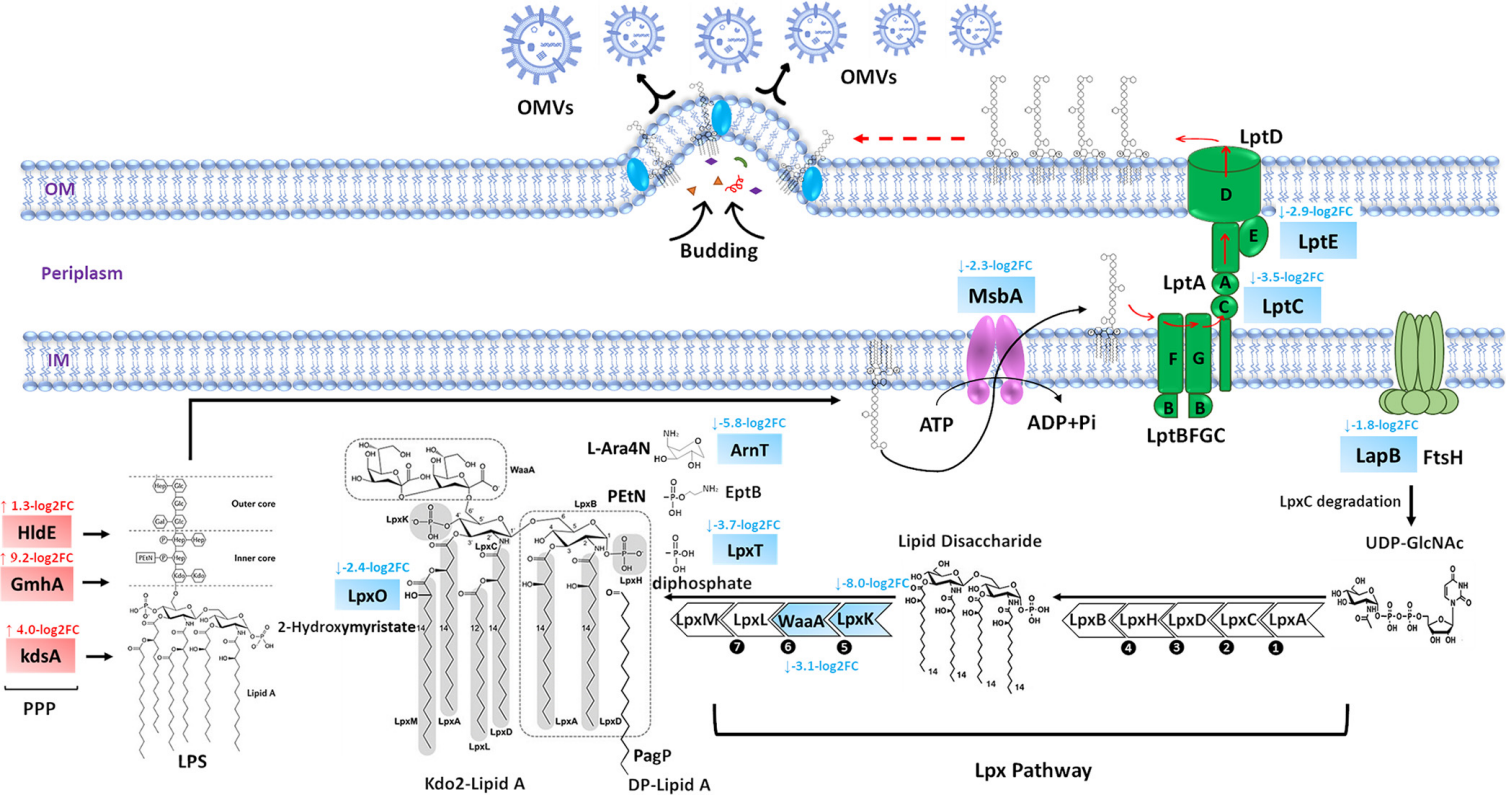
Schematic diagram depicting significantly altered OMV proteins involved in the cytoplasmic LPS biosynthetic steps, LPS periplasmic transport and assembly in the polymyxin-susceptible K. pneumoniae ATCC 700721 following polymyxin B treatment. OM, outer membrane; IM, inner membrane. Blue rectangles, significantly inhibited proteins; red rectangles, significantly increased proteins.

Biosynthetic proteins from the LPS biosynthesis pathway were significantly perturbed in the OMVs from the polymyxin-susceptible strain following polymyxin B treatment, whereas in the OMVs from the polymyxin-resistant strain the LPS biosynthetic proteins were marginally altered (Fig. 2; Table S1 in the supplemental material). The 2-dehydro-3-deoxyphosphooctonate aldolase (KdsA), phosphoheptose isomerase (GmhA), and bi-functional ADP-l-glycero-d-manno-heptose synthase (HldE) were significantly upregulated in the OMVs of polymyxin-susceptible ATCC 700721 following polymyxin B treatment (log_2_ FC = 4.0, 9.2, and 1.3, respectively; Fig. 2; Table S1), whereas in the polymyxin-resistant OMVs, only 2-dehydro-3-deoxyphosphooctonate aldolase (KdsA) was significantly increased following polymyxin B treatment (Table S1). The 2-dehydro-3-deoxyphosphooctonate aldolase is responsible for linking lipid A and core oligosaccharides through synthesis of Kdo (30). In most Gram-negative bacteria, bi-functional ADP-l-glycero-d-manno-heptose synthase and phosphoheptose isomerase proteins are involved in the formation of a key component of the LPS core domain as well as enhancing the bacterial tolerance toward cell-wall-damaging agents (31, 32). It has been shown that the mutation of a gene encoding phosphoheptose isomerase in Gram-negative bacteria results in a compromised outer membrane, leading to a loss of protection normally provided by LPS and increased susceptibility to polymyxins (33).

10.1128/msphere.00537-22.4 TABLE S1 Significantly impacted proteins in polymyxin-susceptible K. pneumoniae ATCC 700721 and polymyxin-resistant K. pneumoniae ATCC 700721R following polymyxin B treatment (>1.0-log_2_-FC, *P ≤ *0.05; FDR ≤ 0.05). Download Table S1, DOCX file, 0.04 MB. Copyright © 2023 Hussein et al. 2023 Hussein et al. https://creativecommons.org/licenses/by/4.0/ This content is distributed under the terms of the Creative Commons Attribution 4.0 International license.

Two essential enzymes of the Lpx pathway (responsible for the synthesis of the Kdo2-lipid A moiety of lipid A) (34), namely, Kdo transferase (WaaA) and tetraacyldisaccharide 4′-kinase (LpxK), were perturbed only in the polymyxin-susceptible OMVs following polymyxin treatment (log_2_FC = −3.1 and −8.0, respectively; Fig. 2; Table S1). Kdo transferase catalyzes the transfer of Kdo to the lipid A precursor of LPS (35), while tetraacyldisaccharide 4′-kinase mediates the sixth step of lipid A biosynthesis by catalyzing phosphorylation of tetraacyldisaccharide-1-phosphate (DSMP) at the 4′ position yielding lipid IV_A_ (36). Finally, the abundance of the LPS assembly protein, LapB, an essential protein that governs the proteolytic activity of FtsH toward LpxC, was substantially decreased in the OMVs of the susceptible strain following polymyxin B treatment (log_2_FC = −1.8) (Fig. 2; Table S1).

The ABC (ATP Binding Cassette) transporter MsbA that transports nascent LPS in a “trap and flip” process, was significantly decreased in the OMVs of the polymyxin-susceptible strain following polymyxin treatment (log_2_FC = −2.3). Moreover, a significant decline in the abundance of two essential proteins, namely, LptC (log_2_FC = −3.5), and LptE (log_2_FC = −2.9), of the Lpt molecular machinery (a unique device that exports LPS across the periplasm to the cell surface) (37), was observed in the OMVs of the polymyxin-susceptible strain following polymyxin treatment (Fig. 2; Table S1).

A significant decrease in the levels of LPS-modifying enzymes was evident mainly in the OMVs of the polymyxin-susceptible strain following polymyxin B treatment. In particular, a marked decrease in the undecaprenyl phosphate-alpha-l-Ara4N transferase (ArnT) was observed in OMVs of both strains (log_2_FC: resistant = −1.1; susceptible = −5.8) (Fig. 2; Table S1). This transferase enzyme catalyzes the transfer of the L-Ara4N moiety to lipid A, an important step for developing polymyxin resistance (15). The LPS-modifying enzyme lipid A 1-diphosphate synthase (LpxT) was markedly decreased (log_2_FC = −3.7) in the polymyxin-susceptible OMVs after polymyxin treatment (Fig. 2; Table S1). Lipid A 1-diphosphate synthase catalyzes phosphorylation of the 1-phosphate of lipid A to form 1-diphosphate (1-PP); this modification increases the net negative charge of lipid A and thereby diminishes the efficiency of the cationic phosphoethanolamine (PEtN) modification. This finding would suggest that the bacteria jettison the enzyme from the OM via OMVs to decrease its abundance and negative impact on PEtN-mediated polymyxin resistance (38, 39). Furthermore, an inner membrane enzyme that catalyzes the oxygen-dependent formation of 2-hydroxymyristate-modified lipid A, namely, acyl-hydroxylase (LpxO), was significantly decreased (log_2_FC = −2.4) in the OMVs of the polymyxin-susceptible strain following polymyxin B treatment (Fig. 2; Table S1). Notably, it has previously been reported that 2-OH myristate modifications of lipid A in pathogenic Gram-negative bacteria Salmonellae promote resistance to CAMPs (e.g., polymyxins and magainin-like peptide pGLa) (40).

The decreased abundance of LPS-modifying enzymes (i.e., ArnT, LpxT, and LpxO) following polymyxin exposure, especially in the OMVs of the polymyxin-susceptible strain, appears to be counterintuitive. However, this could be construed as a mechanism whereby the bacteria generate OMVs decorated with unmodified LPS that is a more attractive target than the bacterial outer membrane that is decorated with modified LPS, resulting in negatively charged OMVs that are more effective in shielding the bacterium and acting as decoys against polymyxin treatment (29, 41 to 43).

### *O*-antigen nucleotide sugar biosynthesis.

The *O*-antigen of Klebsiella spp. comprises repeating oligosaccharide subunits linked to the core antigen of LPS, and the genetics of its biosynthesis in K. pneumoniae is governed by the WB gene clusters (44, 45). In K. pneumoniae, longer *O*-antigen length is linked to increased polymyxin resistance and virulence (44, 46). Proteins of *O*-antigen nucleotide sugar biosynthesis were significantly altered in both polymyxin-susceptible and resistant strain OMVs following treatment (Table S1). In the polymyxin-susceptible strain, six essential proteins involved in *O*-antigen nucleotide sugar biosynthesis underwent a significant overrepresentation in the OMV subproteome following polymyxin treatment, namely, UTP-glucose-1-phosphate uridylyltransferase (GalU), UDP-galactose 4-epimerase (GalE), UDP-glucose dehydrogenase (Ugd), dTDP-4-dehydrorhamnose reductase, glucose-1-phosphate thymidylyltransferase, and dTDP-glucose 4,6-dehydratase (>2.0-log_2_FC, *P ≤ *0.05; FDR ≤ 0.05) (Table S1). Notably, the *O*-antigen export nucleotide binding domain was significantly downregulated in OMVs of the polymyxin-susceptible strain (log_2_FC = −2.64; Table S1).

Polymyxin B treatment caused a marked increase in the levels of four *O*-antigen biosynthetic proteins in the polymyxin-resistant OMV subproteome, including UTP-glucose-1-phosphate uridylyltransferase, UDP-galactose 4-epimerase, UDP-glucose dehydrogenase, and dTDP-4-dehydrorhamnose reductase (>1.0-log_2_FC, *P ≤ *0.05; FDR ≤ 0.05) (Table S1). In Gram-negative bacteria, UDP-glucose dehydrogenase catalyzes the oxidation of UDP-glucose to produce UDP-glucuronic acid, which is required for biosynthesis of many bacterial surface glycostructures, including Ara4N moiety, and secreted exopolysaccharides, which enable the pathogen to develop resistance toward polymyxins and other cationic antimicrobial peptides (47, 48). UDP-glucuronic acid is also an essential substrate for biosynthesis of the *O*-antigen, a component of the outer membrane LPS, and capsular polysaccharides (CPSs) (49). These extracellular polysaccharides are often critical virulence factors that allow the bacteria to invade and colonize the tissue of the host as well as protect the bacteria from the host immune system (e.g., phagocytosis) (50).

### Two-component systems and cationic antimicrobial peptide (CAMPs) resistance.

The PmrA/PmrB two-component systems (TCS) in Gram-negative bacteria are the major regulator of LPS modifications that confer polymyxin resistance (51). PmrD is a small basic protein that serves as the nexus between the PhoP-PhoQ and PmrA-PmrB TCSs (52). PhoP controls the activation of PmrD, which can then bind to PmrA and prolong its phosphorylation state, eventually activating the expression of the PmrA-PrmB (collectively PmrAB) system to promote LPS cationic modifications and resistance to polymyxin (39).

In the OMVs of the polymyxin-susceptible strain, seven crucial components of the OmpR protein family underwent a significant downregulation following polymyxin B treatment, namely, sensor protein PhoQ, phosphate-binding protein PstS, acid phosphatase PhoC, two-component sensor protein CpxA, multidrug resistance protein MdtA, and aerobic respiration control sensor protein (arcB) (>−2.0-log_2_FC, *P ≤ *0.05; FDR ≤ 0.05) (Table S1), whereas the OmpR family negative response regulator acrA displayed a marked upregulation in response to polymyxin treatment (log_2_FC = 6.4). In the OMVs from the polymyxin-resistant strain, only three key proteins of the OmpR family underwent significant downregulation following polymyxin B treatment, namely, alkaline phosphatase PhoA (log_2_FC = −3.6), phosphate-binding protein PstS (log_2_FC = −3.6), and putative outer membrane protein SilC (log_2_FC = −2.3) (Table S1).

It is well acknowledged that the presence of CAMPs can activate PhoQ and thereby stimulate PhoP by phosphorylation, which in turn upregulates the PmrA-PmrB system. The latter TCS is known to confer resistance to polymyxins in K. pneumoniae (53). PhoQ loss-of-function mutations are also associated with a high level of polymyxin resistance in Gram-negative bacteria (54). Importantly, the downregulation of multidrug resistance protein MdtA in the OMVs of the susceptible strain might be an indirect result of the response to polymyxin B treatment, as cyclic AMP receptor protein was significantly upregulated in the OMVs of the susceptible strain and this protein is known for its ability to suppress multidrug efflux pumps (55). The OMVs of the polymyxin-susceptible strain also displayed a marked downregulation of six critical members of the NarL family, namely, outer membrane lipoprotein RcsF, cryptic nitrate reductase 2 alpha subunit, cryptic nitrate reductase 2 beta subunit, fumarate reductase flavoprotein subunit, and succinate dehydrogenase iron-sulfur subunit (>−2.0-log_2_FC, *P ≤ *0.05; FDR ≤ 0.05) (Table S1), whereas the NarL family transcriptional regulatory protein RcsB displayed a marked upregulation (log_2_FC = 5.5) following polymyxin treatment. In addition to PmrA/PmrB, we detected significant perturbations of proteins from various other TCS in the OMVs of the polymyxin-susceptible strain, which were marginally perturbed in the OMVs of the polymyxin-resistant strain (Table S1). In the OMVs of the polymyxin-susceptible strain, these TCS proteins included the Zn-binding periplasmic protein (log_2_FC = −7.9), ATP-binding protein of glutamate (log_2_FC = −5.9), glutamate/aspartate transport protein (log_2_FC = −6.9), cyclic AMP receptor protein (log_2_FC = 5.1), cytochrome oxidase bd-II (log_2_FC = −12.4), protein tyrosine phosphatase (log_2_FC = 3.2), and pectinesterase (log_2_FC = −3.2) (Table S1). Only three non-PmrA/PmrB TCS proteins from the OMV subproteome of the polymyxin-resistant strain were differentially expressed, including ATP-binding protein glutamate (log_2_FC = −2.5), cyclic AMP receptor protein (log_2_FC = 1.0) and pectinesterase (log_2_FC = −1.3) (Table S1). The OMV subproteome in both strains underwent significant downregulation of five essential proteins involved in CAMP resistance in response to polymyxin B treatment, namely, undecaprenyl phosphate-alpha-l-Ara4N transferase (log_2_FC = −1.1, resistant; log_2_FC = −5.8, susceptible), copper homeostasis protein (log_2_FC = −1.8, resistant; log_2_FC = −7.6, susceptible), *N*-acetylmuramoyl-l-alanine amidase (log_2_F = −4.90, resistant; log_2_FC = −6.0, susceptible), thiol:disulfide interchange protein (log_2_FC = −3.4, resistant; log_2_FC = −12.7, susceptible), and peptidyl-prolyl *cis-trans* isomerase (log_2_FC = −3.4, resistant; log_2_FC = −9.7, susceptible) (Table S1). Overproduction of NlpE, an outer membrane lipoprotein, induces the Cpx system (e.g., CpxA), which sequentially mediates the bacterial adhesion and repairs cell envelope proteins (56). The two-component sensor protein CpxA was significantly downregulated only in the susceptible strain OMVs (log_2_FC = −2.8), but not in the OMVs of the polymyxin resistance strain. Notably, the peptidoglycan biosynthetic enzyme *N*-acetylmuramoyl-l-alanine amidase, and three folding chaperones, namely, thiol:disulfide interchange protein, peptidyl-prolyl *cis*-*trans* isomerase, and putative enzyme ycfS (proteins downstream of the Cpx pathway), underwent a profound decline only in the polymyxin-susceptible OMVs strain (Table S1). Intriguingly, PhoQ and acridine efflux pump, a membrane fusion protein and part of the resistance-nodulation-division family of proteins (57), was markedly downregulated in the OMVs of the polymyxin-susceptible strain (log_2_FC = −1.4 and −2.2, respectively) (Table S1). Moreover, other peptide transport proteins commonly involved in resistance to CAMPs and bacterial virulence, namely, peptide transport proteins sapA (log_2_FC = −2.5) and sapF (log_2_FC = −2.7) (58), underwent a significant decline in their abundance, but only in the OMVs of the polymyxin-susceptible strain (Table S1). It has been shown that an increase in the expression of ABC transporters encoded by the *sapABCDFZ* operon enhances the development of CAMP bacterial resistance by transferring the CAMPs into the cytoplasm for intracellular proteolytic degradation (58).

### β-lactam resistance.

Polymyxin B treatment produced a significant downregulation in the levels of key proteins involved in β-lactam resistance of the OMV subproteomes of both strains, particularly the polymyxin-susceptible strain (>−1.0-log_2_-FC, *P ≤ *0.05; FDR ≤ 0.05), namely, TEM-1 β-lactamase Class A, RND multidrug efflux systems, namely, acridine efflux pump, bifunctional penicillin-binding protein 1a, and periplasmic oligopeptide-binding protein (Table S1). Notably, the OMVs of the resistant strain displayed a significant downregulation in the abundance of class A extended-spectrum β-lactamase SHV-12 (log_2_FC = −2.5) and TEM-1 β-lactamase Class A (log_2_FC = −2.5) in response to polymyxin B treatment (Table S1). Similarly, the levels of periplasmic oligopeptide-binding protein in the OMVs of the resistant strain were downregulated in response to polymyxin B treatment (Table S1).

### Peptidoglycan biosynthesis.

**FIG 3:**
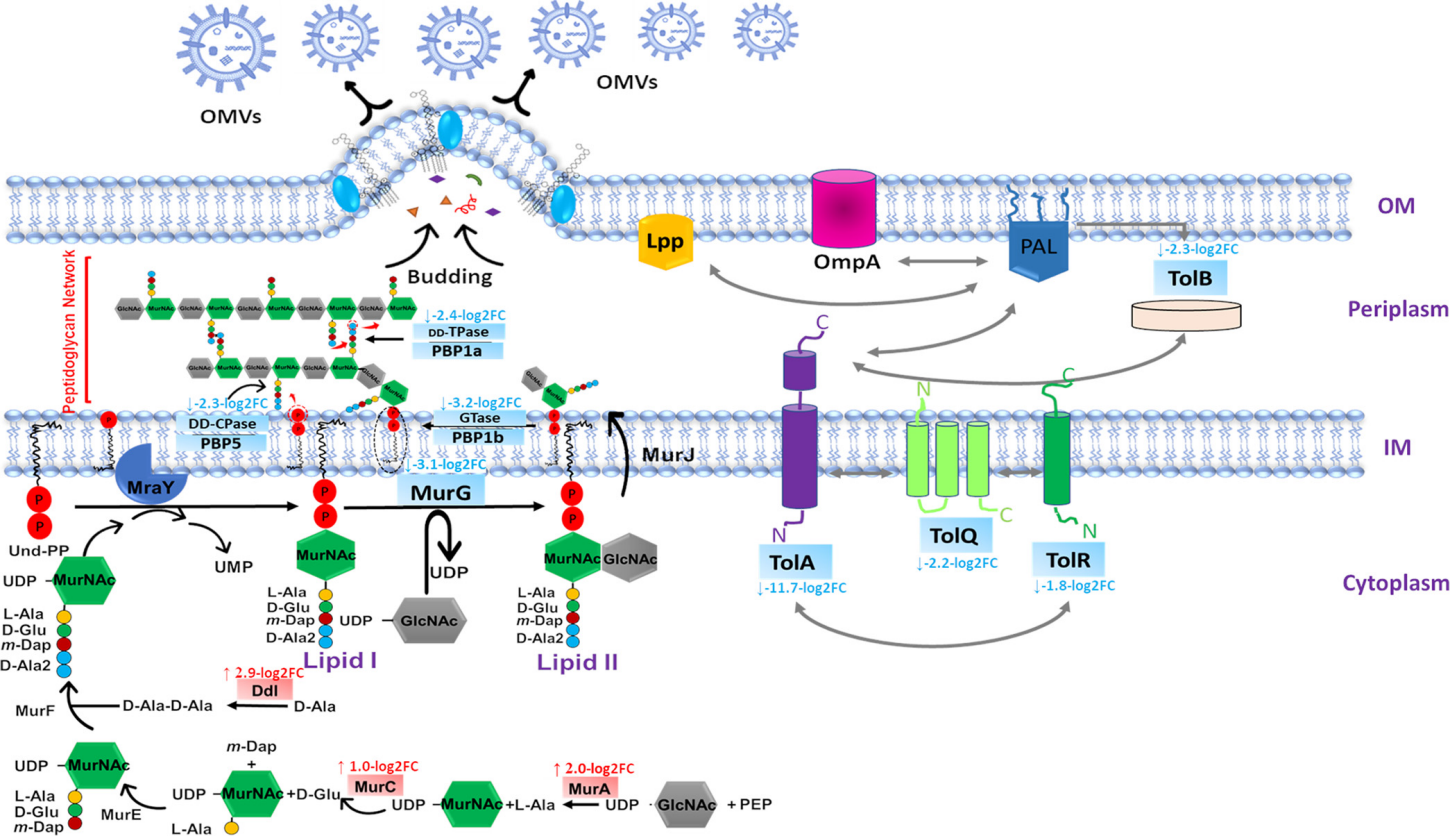
Schematic diagram shows the essential proteins involved in the peptidoglycan biosynthesis and Tol-Pal system in the OMVs of polymyxin-susceptible K. pneumoniae ATCC 700721 significantly altered following polymyxin B treatment. Blue rectangles, significantly inhibited proteins; red rectangles, significantly increased proteins.

Fundamental proteins involved in peptidoglycan biosynthesis underwent significant alterations, particularly in the OMVs of the polymyxin-susceptible strain (Fig. 3; Table S1). UDP-*N-acetylglucosamine* 1-carboxyvinyltransferase (MurA), UDP-*N*-acetylmuramate-l-alanine ligase (MurC), and d-alanine-d-alanine ligase (Ddl) were among the essential enzymes of peptidoglycan biosynthesis that were significantly upregulated (log_2_FC = 2.0, 1.0, and 2.9, respectively; Fig. 3; Table S1), whereas, undecaprenyl-PP-MurNAc-pentapeptide-UDPGlcNAc GlcNAc transferase (MurG), bifunctional penicillin-binding protein 1a (PBP1a), penicillin-binding protein 1b (PBP1b), and d-ala-d-ala carboxypeptidase (PBP 5/DD-CPase) were all significantly downregulated (log_2_FC = −3.1, −2.4, −3.2, and −2.3, respectively; Fig. 3; Table S1). Furthermore, in the OMVs of the polymyxin-susceptible strain, a set of membrane-bound PBPs that mediate the final stages of peptidoglycan synthesis were significantly downregulated (59), namely, penicillin-binding protein activator LpoB (PBP activator LpoB), carboxy-terminal protease for penicillin-binding protein 3 (PBP 3), endolytic murein transglycosylase protein (YceG), penicillin-insensitive murein endopeptidase (MepA), and d-alanyl-d-alanine carboxypeptidase penicillin-binding protein 6 (DacC) (log_2_FC = −9.3, −5.7, −5.7, −7.4, and −5.3, respectively; Table S1). Notably, endolytic peptidoglycan transglycosylase (RlpA), which functions to repair aberrantly formed peptidoglycan (60), was significantly increased (log_2_FC = 3.7). In the OMVs of the resistant strain, polymyxin treatment induced marked perturbations in d-ala-d-ala carboxypeptidase (PBP5), carboxy-terminal protease for penicillin-binding protein 3, and endolytic peptidoglycan transglycosylase (log_2_FC = 1.0, −1.9, and −3.8, respectively; Table S1). Intriguingly, the Tol-Pal system and other related proteins that mediate peptidoglycan synthesis and outer membrane constriction during cell division (61) were significantly downregulated, particularly in the susceptible strain OMV subproteome (Table S1). These included TolB (log_2_FC = −2.9, resistant; log_2_FC = −2.3, susceptible), TolQ (log_2_FC = −2.2, susceptible), TolR (log_2_FC = −1.8, susceptible), and the cell division coordinator CpoB (log_2_FC = −3.7, resistant; log_2_FC = −5.4, susceptible). The levels of TolA were elevated in the resistant strain OMVs (log_2_FC = 4.4), whereas its abundance was markedly decreased in the OMVs of the susceptible strain (log_2_FC = −11.7; Fig. 3; Table S1).

Overall, there appears to be a significant reduction in the peptidoglycan biosynthetic machinery within the OMVs of the susceptible strain, whereas they were mildly perturbed in the OMVs of the polymyxin resistant strain.

### Protein export and outer membrane proteins (OMPs) complex biosynthesis.

The Gram-negative protein export system is composed of sophisticated protein machinery that efficiently exports intracellular proteins, such as the twin-arginine translocation (Tat) system (transports folded proteins), Sec pathway (general secretory pathways for unfolded preproteins), and YidC (folding and assembly of membrane proteins) (62). The Sec machinery comprises a complex of interrelated cytoplasmic membrane pathways, namely, SecA, SecB, SecYEG, SecDF, YajC, YidC, and SRP pathways (63). Secretion of various protein components is often paramount for bacterial pathogenicity and virulence mechanisms like biofilm formation (64). In response to the polymyxin B treatment, the Sec-dependent pathway and Tat system proteins were minimally perturbed in OMVs subproteome of the resistant strain, while they underwent a greater alteration in their abundance in the OMVs proteins of the susceptible strain (Table S1).

**FIG 4:**
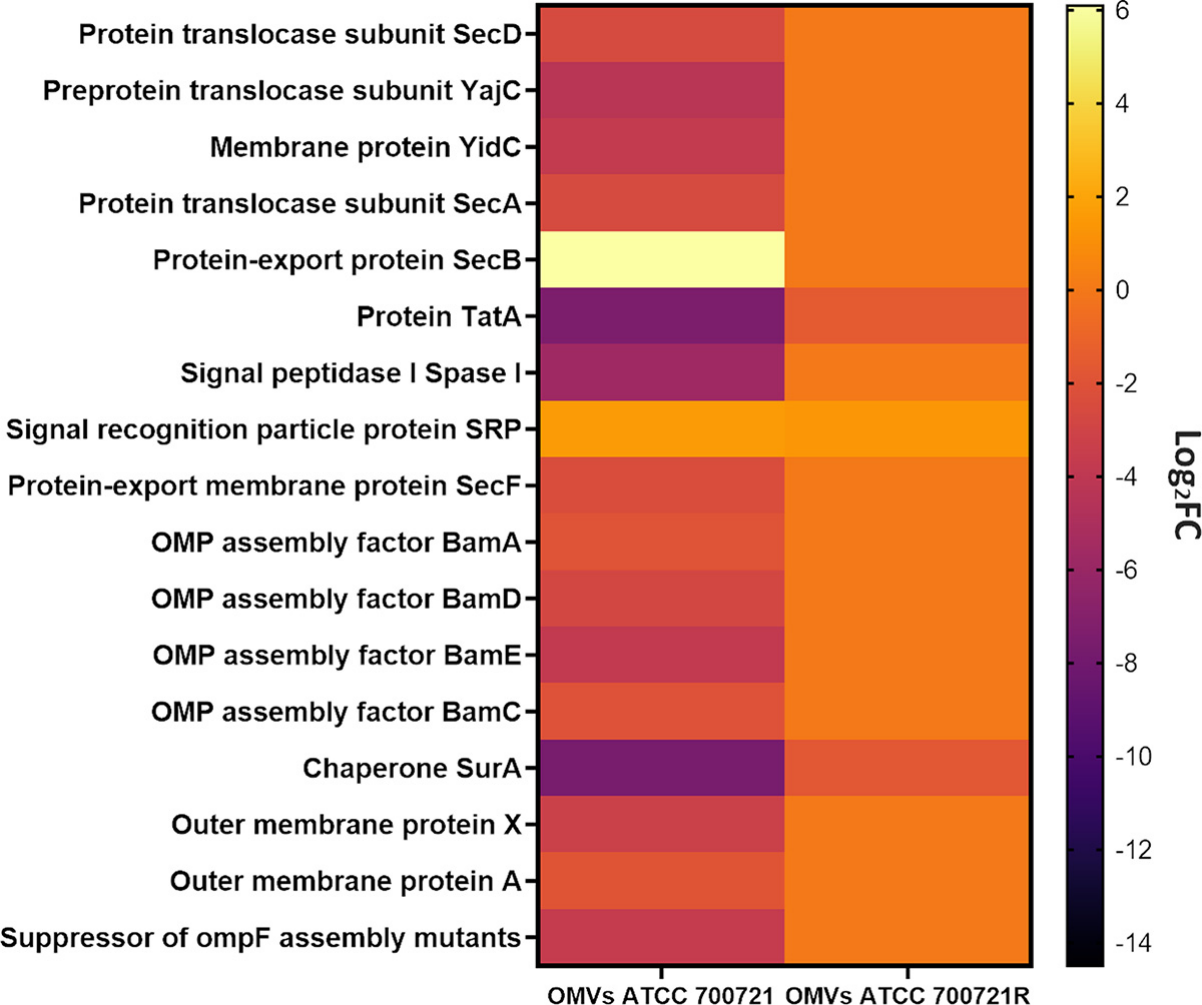
Heatmap displays the main proteins involved in protein export and outer membrane proteins (OMPs) complex biosynthesis in the OMVs of polymyxin-susceptible K. pneumoniae ATCC 700721 and its polymyxin-resistant derivative ATCC 700721R significantly altered following polymyxin B treatment.

In the susceptible OMVs subproteome, there was a significant increase in the levels of SecB (log_2_FC = 6.0) and signal recognition particle protein SRP (log_2_FC = 1.5) following polymyxin B treatment (Fig. 4). In contrast, a substantial decline in the abundance of several key components of the Sec pathway was observed following polymyxin B treatment, namely, SecA, SecD, SecF, YajC, and YidC (>−2.0-log_2_FC, *P ≤ *0.05; FDR ≤ 0.05) (Fig. 4; Table S1). Furthermore, an essential integrated subunit of the twin-arginine translocation system TatA protein and Signal peptidase I (Spase I), a vital enzyme required for the bacterial survival and essential for the translocation of preproteins from the cytoplasmic to periplasmic location (65), were significantly downregulated in response to polymyxin B treatment (log_2_FC = −7.4 and −5.7, respectively) (Fig. 4; Table S1).

In the OMVs of the polymyxin-resistant strain, an upregulation of signal recognition particle protein SRP (log_2_FC = 1.3) and downregulation of TatA protein were observed in response to polymyxin B treatment (log_2_FC = −1.5; Table S1). Moreover, polymyxin B treatment caused a marked reduction in the levels of four fundamental proteins of a highly conserved multiprotein Bam complex in the OMVs of the polymyxin-susceptible strain, including OMP assembly factor BamA, BamC, BamD, and BamE (>−2.0-log_2_FC, *P ≤ *0.05; FDR ≤ 0.05) (Fig. 4; Table S1). These proteins accelerate the folding and membrane integration of β-barrel transmembrane proteins and are involved in a range of functions, including the creation of pores to allow the passage of diverse molecules across the bacterial membrane. Their assembly is thus essential for maintaining the integrity of the cell envelope (66). Notably, the OMVs proteins of both strains had a considerable downregulation of the chaperone protein SurA, a critical enzyme required for protecting OMPs from aggregation in the periplasm (log_2_FC = −7.6 and −1.7, respectively) (Fig. 4; Table S1). It is noteworthy that SurA depletion results in a marked decrease in the outer membrane density (67). The OMVs of the polymyxin-susceptible strain treated with polymyxin B displayed a significant reduction in the abundance of three essential outer membrane proteins, namely, outer membrane protein A, outer membrane protein X, and suppressor of ompF assembly mutants (>−1.0-log_2_FC, *P ≤ *0.05; FDR ≤ 0.05) (Fig. 4; Table S1). The OmpA proteins in K. pneumoniae are major membrane proteins that are crucial for the bacterial pathogenicity (adhesion and invasion), bacterial envelope integrity (via anchoring the outer membrane to the peptidoglycan), and transfer of molecules. Notably, it has been reported that the knockout of *ompA* in K. pneumoniae resulted in a highly polymyxin susceptible phenotype (20). Perturbations in these proteins were not detected in the OMVs of the polymyxin-resistant strain.

### RNA degradation and nucleotide excision/repair.

The RNA degradosome is a multiprotein complex protein machine that mediates the degradation of mRNA (68). The OMVs of both strains exhibited marked perturbations in the levels of principle components of the RNA degradosome in response to polymyxin treatment (Table S1). In the OMVs of the polymyxin-susceptible strain, the abundance of RNase E (RNase E), ATP-dependent RNA helicase RhlB, ATP-dependent RNA helicase DeaD, polyphosphate kinase (PKK), and poly(A) polymerase I (PAP I) all significantly decreased following polymyxin B treatment (>−1.0-log_2_FC, *P ≤ *0.05; FDR ≤ 0.05) (Table S1). In contrast, three key RNA degradosome constituents were significantly increased in response to polymyxin B treatment, namely, enolase (log_2_FC = 7.9), ATP-dependent 6-phosphofructokinase (log_2_FC = 4.7), and the 60-kDa GroEL chaperonin (log_2_FC = 6.0) (Table S1). On the other hand, in the OMVs of the polymyxin-resistant strain, polymyxin B treatment induced a considerable increase in the abundance of five principal components of the RNA degradosome, namely, polyphosphate kinase, 60-kDa GroEL chaperonin, enolase, RNase E, and ATP-dependent RNA helicase RhlB (>1.0-log_2_FC, *P ≤ *0.05; FDR ≤ 0.05) (Table S1). RNA-binding protein Hfq (an RNA degradosome regulator that binds small regulatory RNAs and mRNAs to mediate mRNA translation in response to the bacterial envelope stressors such as polymyxins) was significantly overexpressed (log_2_FC = 5.7) (Table S1) (68). Notably, there was also a significant upregulation in critical proteins involved in the nucleotide excision and repair, namely, UvrABC repair system (log_2_FC = 1.1) and DNA helicase (log_2_FC = 4.7) (69). In the OMVs of polymyxin-susceptible strain, the UvrABC repair system was significantly downregulated following polymyxin B treatment (log_2_FC = −2.9) (Table S1). Taken together, these findings would suggest that compared to its susceptible pair, resistant strain may have an enhanced ability to withstand DNA lesions.

### Quorum sensing (QS).

Gram-negative bacteria employ QS by secretion of *N*-acyl homoserine lactones (AHLs), sometimes in conjunction with TCS, to coordinate pathogenic behaviors such as virulence factor expressions, biofilm formation, and motility (70). Although the OMV subproteomes in both strains displayed significant perturbations in the quorum-sensing proteins in response to polymyxin B treatment, the extent of the perturbations observed in the polymyxin-susceptible strain were significantly greater. In the OMV subproteome of the polymyxin-susceptible strain, the abundance of autoinducer 2-binding protein LsrB, three oligopeptide transport proteins (periplasmic oligopeptide-binding protein, oligopeptide transporter ATP-binding component, and oligopeptide transport protein), membrane protein insertase YidC, periplasmic murein tripeptide (l-Ala-gamma-d-Glut-*m*-DAP) permease, protein translocase subunit SecA, preprotein translocase subunit YajC, putative ABC transporter periplasmic binding protein, and high-affinity branched-chain amino acid transport protein, all were significantly downregulated in response to polymyxin treatment (>−2.0-log_2_FC, *P ≤ *0.05; FDR ≤ 0.05) (Table S1). Similarly, but to a lesser extent, five essential proteins of a quorum-sensing system underwent a considerable downregulation in the OMVs of the polymyxin resistant strain after polymyxin B treatment, namely, periplasmic oligopeptide-binding protein, permease, oligopeptide transport protein, high-affinity branched-chain amino acid transport protein, and putative ABC transporter periplasmic binding protein (>−1.0-log_2_FC, *P ≤ *0.05; FDR ≤ 0.05) (Table S1). Perturbations were also evident in systems involved in biofilm formation. Notably, there was a significant decrease in the levels of autoinducer 2-binding protein LsrB component of the LuxS-dependent autoinducer AI-2 system in the polymyxin-susceptible OMVs, whereas a marked elevation in the abundance of 3-hydroxy-5-phosphonooxypentane-2,4-dione thiolase was observed following polymyxin B treatment (log_2_FC = 8.4) (Table S1). Furthermore, the protein-export machinery SecB protein levels were markedly upregulated in the polymyxin-susceptible OMVs after polymyxin B treatment (log_2_FC = 8.4) (Table S1). Cyclic AMP receptor protein, which is involved in biofilm formation and flagellar biosynthesis for motility (71), was significantly upregulated in both strains (log_2_FC = 1.0, resistant; log_2_FC = 5.1, susceptible).

### Conclusions.

Collectively, our findings indicate a potential role for OMVs in neutralizing the antimicrobial action of polymyxins, through the delivery of protein cargo that facilitates resistance and cellular repair processes. In response to polymyxin B exposure, both the susceptible and resistant K. pneumoniae strains responded by modifying their OMV subproteome to include proteins involved in outer membrane remodeling (LPS, *O*-antigen and peptidoglycan biosynthesis), CAMP resistance, β-lactam resistance, RNA degradation, and nucleotide excision/repair and quorum sensing. Overall, this study highlights the importance of OMVs as “molecular mules” for the intercellular transmission and delivery of resistance and cellular repair factors in the bacterial response to polymyxin B treatment.

## MATERIALS AND METHODS

### Reagents.

All chemicals were purchased from Sigma-Aldrich at the highest research grade, with the exception of the ultrapure water (Fluka, Castle Hill, NSW, Australia), Tris (ICN biochemicals, Castle Hill, NSW, Australia), the precast-SDS gels (NuSep Ltd., Lane Cove, NSW, Australia), and polymyxin B (Beta pharma, Shanghai, China). Stock solutions of polymyxin B (10 mg/L) were prepared in Milli-QTM water (Millipore, North Ryde, NSW, Australia) and filtered through 0.22-μm syringe filters (Sartorius, Melbourne, Vic, Australia).

### Bacterial isolates and growth conditions.

K. pneumoniae ATCC 700721 (polymyxin B MIC = 0.5 mg/L) and its paired polymyxin-resistant strain K. pneumoniae ATCC 700721R (polymyxin B MIC ≥128 mg/L) were employed in this study. A polymyxin-resistant variant of K. pneumoniae ATCC 13883 (designated 13883R) was generated as previously described (72). Bacteria were stored at −80°C in tryptone soya broth (TSB, Oxoid Australia, West Heidelberg, Victoria, Australia). One day prior to each experiment, bacteria were streaked onto nutrient agar plates (Medium Preparation Unit, University of Melbourne, Victoria, Australia). Overnight cultures were prepared by inoculating one colony in 5 mL of cation-adjusted Mueller-Hinton broth (CAMHB, Oxoid), from which a 1-in-100 dilution was made in fresh broth to prepare midlogarithmic cultures with the optimal OD600 nm ~0.5. All broth cultures were incubated at 37°C in a rotary shaker (180 rpm).

### Measurements of MICs.

The European Committee on Antimicrobial Susceptibility Testing (EUCAST) clinical breakpoints for polymyxin B for Enterobacteriaceae (e.g., K. pneumoniae) are as a susceptible breakpoint of ≤2 mg/L and a resistant breakpoint >2 mg/L (73). MIC determinations for all isolates according to CLSI guidelines using broth microdilution method were performed using three replicates (74). The microtiter plates were inoculated with 100 μL of bacterial suspension (10^6^ CFU per mL) and 100 μL of serial dilutions of the polymyxin B. Cell viability was determined by sampling wells at polymyxin concentrations greater than the MIC. These samples were serially diluted in 0.9% saline solution and plated onto nutrient agar. After incubation at 37°C for 20 h, viable colonies were counted on these plates using a plate counter (Synbiosis ProtoCOL 3, Thermo-Fischer Scientific, Melbourne, Australia).

### Isolation of outer membrane vesicles (OMVs).

Midlogarithmic cultures (4 L) of each isolate were grown, polymyxin B was added to the culture volume at a final concentration of 2 mg/L, and cell-free supernatants were collected through centrifugation (15 min at 10,000 × *g*, 4°C). The OMV-containing supernatants were filtered through 0.22-μm membrane to remove any remaining cell debris, then concentrated through a tangential filtration concentrator unit (Pall Life Science, Ann Arbor, MI) and collected using 100 kDa Pellicon filtration cassettes (Millipore, Melbourne, Australia). A portion of the supernatant was plated for growth on agar plates overnight at 37°C to make sure that the supernatant was free of bacterial cells. OMVs in the cell-free supernatants were then pelleted down by ultracentrifugation at 150,000 × *g* for 2 h at 4°C in a Beckman Ultracentrifuge (SW28 rotor). Purified OMVs were concentrated and resuspended in 1 mL of sterile phosphate-buffered saline (PBS). Samples were analyzed by 1D-SDS-PAGE and the protein concentrations were determined using the Bio-Rad protein assay according to the manufacturer’s protocol (Bio-Rad, Melbourne, Australia).

### Ultra-high-performance liquid chromatography coupled with mass spectrometry.

For all experiments, an Ultimate3000 ultra-high-pressure liquid chromatography (UHPLC) system (Dionex, Castle Hill, Sydney) was used equipped with a ternary low-pressure mixing gradient pump (LPG-3600), a membrane degasser unit (SRD-3600), a temperature-controlled pulled-loop auto-sampler (WPS-3000T), and a temperature-controlled column oven with flow manager (FLM-3100). The UHPLC experiments were performed using the “preconcentration” setup under the following conditions: nano-column C_18_ PepMap100, 75 μm ID × 150 mm, 3 μm, 100 Ǻ; mobile phase A: 99.9% water + 0.1% FA (vol/vol, formic acid); mobile phase B: 20/80 water/acetonitrile (ACN) (vol/vol) + 0.08% formic acid; flow rate nano-column, 400 nL/min; gradient, 2 to 40% B over 45 min, 90% B for 5 min, 4% B for 30 min; loop size, 5 μL; injection volume, 4 μL (FullLoop) by User Defined Program. The oven temperature was set to 35°C. Collision-induced dissociation (CID) experiments for peptide identification were performed using an AmaZon ETD Ion Trap (Bruker Daltonik GmbH, Australia) equipped with an online nano-sprayer spraying from a 0.090 mm inside diameter (i.d.) and 0.02 mm i.d. fused silica capillary. Fine tuning was achieved using the smart parameter setting option (SPS) for 900 *m/z*, compound stability 60%, and trap drive level at 100% in normal mode resulted in the following mass spectrometric parameters: dry gas temperature, 180°C; dry gas, 4.0 L min^−1^; nebulizer gas, 0.4 bar; electrospray voltage, 4,500 V; high-voltage end-plate offset, −200 V; capillary exit, 140 V; trap drive, 57.4; funnel 1 in 100 V, out 35 V and funnel 2 in 12 V, out 3.3 V; immunofluorescent cell count (ICC) target, 500,000; maximum accumulation time, 50 ms. The sample was measured with the enhanced scan mode at 8,100 *m/z* per second (which allows monoisotopic resolution up to four charge stages), polarity positive, scan range from 100 to 3,000 *m/z*, 5 spectra averaged, and rolling average of 2. Acquired tandem mass spectra were processed in Data Analysis 4.0; deconvoluted spectra were further analyzed with BioTools 3.2 software and submitted to Mascot database search (Mascot 2.2.04, Swissprot database). The species subset was set at K. pneumoniae, parent peptide mass tolerance ±0.4 Da, fragment mass tolerance ±0.4 Da; enzyme specificity trypsin with 2 missed cleavages considered. The following variable modifications have been used: Deamidation (NQ), Oxidation (M), and carbamidomethylation (C).

### Bioinformatics analysis.

The derived MS data sets on the 3D-trap system were combined into protein compilations using the Protein Extractor functionality of Proteinscape 2.1.0 573 (Bruker Daltonics, Bremen, Germany), which conserved the individual peptides and their scores, while combining them to identify proteins with much higher significance than what is achievable using individual searches. To exclude false-positive identifications, peptides with Mascot scores below 40 (selected based on manual evaluation of the MS/MS data of peptides with scores below this number) were rejected. The identified protein sequences were manually validated in BioTools (Bruker Daltonics, Bremen, Germany) on a residue-by-residue basis using the raw data to ensure accuracy. Since this strain (ATCC 700721/MGH78578) has a complete genomic map in UniProt (75), the protein name and KEGG ID for the identified proteins were determined by performing a search in UniProt and cross-referencing using MGH78578 KEGG identifiers (76). The subcellular localization of each protein was predicted using PSORTb 3.0 and Cello V2.5 (77, 78). Furthermore, VirulentPred (http://bioinfo.icgeb.res.in/virulent/) was also employed to screen for bacterial virulence protein sequences of OMV subproteome. Comparative analysis of significantly changed proteins (fold change >2 and *P* value <0.05; FDR ≤0.05) was performed using MaxQuant (version 1.2.2.5) (79). The protein-associated metabolic pathways of deferentially over- and underrepresented proteins were highlighted in KEGG mapper.

10.1128/msphere.00537-22.1 FIG S1 The prediction of subcellular localization of different K. pneumoniae proteins identified using PSORTb 3.0 and Cello V2.5 following polymyxin B treatment. (A) Subcellular localization of identified OMV proteins from ATCC 700721. (B) Subcellular localization of identified OMV proteins from ATCC 700721R. Download FIG S1, TIF file, 2.1 MB. Copyright © 2023 Hussein et al. 2023 Hussein et al. https://creativecommons.org/licenses/by/4.0/ This content is distributed under the terms of the Creative Commons Attribution 4.0 International license.

10.1128/msphere.00537-22.2 FIG S2 Subcellular localization of quantified proteins based on their enrichment or depletion in OMVs following polymyxin B treatment. Significantly up- (red) and downregulated (blue) OMV proteins in polymyxin-susceptible K. pneumoniae ATCC 700721 (A) and polymyxin-resistant K. pneumoniae ATCC 700721R (B). The proteins were sorted according to predicted subcellular localization. C, cytoplasmic; IM, inner membrane; P, periplasmic; OM, outer membrane; E, extracellular. (≥ 1.0 log_2_FC, *P* ≤ 0.05; FDR ≤ 0.05). Download FIG S2, TIF file, 2.0 MB. Copyright © 2023 Hussein et al. 2023 Hussein et al. https://creativecommons.org/licenses/by/4.0/ This content is distributed under the terms of the Creative Commons Attribution 4.0 International license.

10.1128/msphere.00537-22.3 FIG S3 (A) OMV proteins predicted to be associated with the virulence in polymyxin-susceptible K. pneumoniae ATCC700721 and its paired polymyxin-resistant strain following polymyxin B treatment. (B) Venn diagram for the OMVs proteins presumed to be correlated with the virulence in polymyxin-susceptible K. pneumoniae ATCC 700721 and polymyxin-resistant strain (>1.0-log_2_-FC, *P ≤ *0.05; FDR ≤ 0.05). Download FIG S3, TIF file, 2.6 MB. Copyright © 2023 Hussein et al. 2023 Hussein et al. https://creativecommons.org/licenses/by/4.0/ This content is distributed under the terms of the Creative Commons Attribution 4.0 International license.
