# Supplementary material for: Comparative Proteomics of Outer Membrane Vesicles from Polymyxin-Susceptible and Extremely Drug-Resistant Klebsiella pneumoniae
Source: mSphere. 2023 Jan 9;8(1):e00537-22. doi: 10.1128/msphere.00537-22 (PMC9942579; doi:10.1128/msphere.00537-22)
Supplement: TEXT S1 [file msphere.00537-22-s0001.docx]

**Comparative Proteomics of Outer Membrane Vesicles from Polymyxin-Susceptible and Extremely Drug Resistant *Klebsiella pneumoniae***

Maytham Hussein,^1#^ Raad Jasim,^5#^ Hakan Gocol,^1^ Mark Baker,^3^ Varsha J Thombare,^1^ James Ziogas,^1^ Aayush Purohit,^4^ Gauri G. Rao,^4^ Jian Li,^2^* Tony Velkov^1^*

**Affiliations:** ^1^Monash Biomedicine Discovery Institute, Department of Microbiology, Monash University, Clayton, VIC 3800, Australia; ^2^Department of Biochemistry & Pharmacology, School of Biomedical Sciences, Faculty of Medicine, Dentistry and Health Sciences, The University of Melbourne, Parkville, VIC, 3010, Australia;  ^3^Discipline of Biological Sciences, Priority Research Centre in Reproductive Biology, Faculty of Science and IT, University of Newcastle, University Drive, Callaghan NSW, 2308, Australia; ^4^Division of Pharmacotherapy and Experimental Therapeutics, Eshelman School of Pharmacy, University of North Carolina, Chapel Hill, North Carolina, USA; ^5^Department of Pharmacology, College of Pharmacy, University of Babylon, Iraq.

*Corresponding authors: [tony.velkov@unimelb.edu.au](mailto:tony.velkov@unimelb.edu.au) OR [jian.li@monash.edu](mailto:jian.li@monash.edu)

#These authors contributed equally to this work.

**KEYWORDS.** Polymyxin B, OMVs, Proteomics, MDR Gram-negative.

**Short Title:** OMV sub-proteome of polymyxin resistant *Klebsiella pneumoniae*

**S1. Subcellular localisation and virulence prediction of the OMV sub-proteomes**

The OMV sub-proteomes of *K. pneumoniae* ATCC 700721 were mostly cytoplasmic (55-56%) in both strains (**Supplementary Figure S1A and S1B**), followed by inner membrane proteins (22-23%) and periplasmic proteins (14%). Minor OMV proteins were in similar proportions across both strains, namely from the outer membrane (6%) and extracellular secreted components (2%) (**Supplementary Figure S1A and S1B**). Polymyxin B treatment (2 mg/L for 2 h) significantly increased the number of cytoplasmic proteins (246/298) in the OMVs of the susceptible strain, whereas there was a significant downregulation in the levels of proteins from the inner membrane (148/157), periplasmic space (137/144), outer membrane (32/35), and extracellular secreted proteins (7/13) (**Supplementary Figure S2A**). Similarly, polymyxin B treatment induced an upregulation of cytoplasmic proteins (96/100) in the OMVs of the polymyxin resistant strain, whereas the abundance of proteins from the inner membrane (8/15), periplasmic space (84/93), outer membrane (17/19), and extracellular proteins (3/4) all decreased (**Supplementary Figure S2B**). Virulence factor predictions using the VirulentPred algorithm illustrated that there was no significant difference in the number of identified OMV proteins that relate to bacterial virulence in both polymyxin-susceptible (289) and -resistant (294) *K. pneumoniae* strains, following polymyxin B treatment (**Supplementary Figure S3A**). However, 32 unique OMV proteins were identified in the polymyxin resistant strain and 28 unique OMV proteins were identified in the susceptible strain (**Supplementary Figure S3B**).
