# Supplementary material for: Comparative Proteomics of Outer Membrane Vesicles from Polymyxin-Susceptible and Extremely Drug-Resistant Klebsiella pneumoniae
Source: mSphere. 2023 Jan 9;8(1):e00537-22. doi: 10.1128/msphere.00537-22 (PMC9942579; doi:10.1128/msphere.00537-22)
Supplement: TABLE S1 [file msphere.00537-22-s0005.docx]

| ***Lipopolysaccharide (LPS) biosynthesis and its modification*** | | | |
| --- | --- | --- | --- |
|  |  | ***K. pneumoniae* ATCC 700721** | |
| **Protein name** | **Gene** | **Susceptible** | **Resistant** |
| Undecaprenyl phosphate-alpha-L-Ara4N transferase arnT | *arnT* | -5.8 | -1.1 |
| 2-dehydro-3-deoxyphosphooctonate aldolase KdsA | *kdsA* | 4.0 | 1.9 |
| Kdo transferase WaaA | *waaA* | -3.1 | - |
| Tetraacyldisaccharide 4'-kinase LpxK | *lpxK* | -8.0 | - |
| beta-hydroxylase LpxO | *LpxO* | -2.4 | - |
| Lipid A 1-diphosphate synthase LpxT | *LpxT* | -3.7 | - |
| Phosphoheptose isomerase  GmhA | *lpcA(gmhA)* | 9.2 | - |
| bi-functional ADP-L-glycero-D-manno-heptose synthase HldE | *rfaE* | 1.3 | - |
| Lipopolysaccharide assembly protein B LapB | *lapB(yciM)* | -1.8 | - |
| Lipopolysaccharide export system protein LptC | *yrbK* | -3.5 | - |
| LPS-assembly lipoprotein LptE | *rlpB* | -2.9 | - |
| Lipid A export ATP-binding/permease protein MsbA | *msbA* | -2.3 | - |
| ***Peptidoglycan biosynthesis*** | | | |
| UDP-N-acetylglucosamine 1-carboxyvinyltransferase (MurA) | *murA* | 2.0 | -1.1 |
| UDP-N-acetylmuramate-L-alanine ligase (MurC) | *murC* | 1.0 | 1.9 |
| D-alanine-D-alanine ligase Ddl | *ddl* | 2.9 | - |
| Undecaprenyl-PP-MurNAc-pentapeptide-UDPGlcNAc GlcNAc transferase (MurG) | *murG* | -3.1 | - |
| Penicillin-binding protein 1B (PBP-1b) | *mrcB* | -3.2 | - |
| DD-transpeptidase | *mrcA* | -2.4 | - |
| D-alanyl-D-alanine carboxypeptidase (PBP 5) | *dacA* | -2.3 | 1.0 |
| Bifunctional penicillin-binding protein (PBP1a) | *mrcA* | -2.4 | - |
| D-alanyl-D-alanine carboxypeptidase DacC (PBP6) | *dacC* | -5.3 | - |
| Carboxy-terminal protease for penicillin-binding protein 3 (PBP3) | *prc* | -5.7 | -1.9 |
| Penicillin-insensitive murein endopeptidase MepA | *mepA* | -7.4 | - |
| Penicillin-binding protein activator LpoB (PBP activator LpoB) | *lpoB* | -9.3 | - |
| Endolytic murein transglycosylase YceG | *yceG* | -5.7 |  |
| Endolytic peptidoglycan transglycosylase RlpA | *rlpA* | 3.7 | -3.8 |
| Tol-Pal system protein TolA | *KPN_01758* | -11.7 | 4.4 |
| Protein TolB | *tolB* | -2.3 | -2.9 |
| Tol-Pal system protein TolR | *tolR* | -1.8 | - |
| Tol-Pal system protein TolQ | *tolQ* | -2.2 | - |
| Cell division coordinator CpoB | *ybgF* | -5.4 | -3.7 |
| ***O-antigen nucleotide sugar biosynthesis*** | | | |
| UTP-glucose-1-phosphate uridylyltransferase (GalU) | *galU* | 5.0 | 1.8 |
| UDP-galactose 4-epimerase (GalE) | *galE* | 2.5 | 1.2 |
| UDP-glucose dehydrogenase (Ugd) | *ugd* | 2.6 | 1.0 |
| dTDP-4-dehydrorhamnose reductase | *rmlD* | 2.5 | 1.0 |
| Glucose-1-phosphate thymidylyltransferase | *rmlA* | 2.5 | - |
| dTDP-glucose 4,6-dehydratase | *rmlB* | 2.6 | - |
| O-antigen export-NBD component | *KPN-02485* | -2.64 | - |
| ***Protein export and Outer membrane proteins (OMPs) complex biosynthesis*** | | | |
| Protein translocase subunit SecD | *secD* | -2.6 | - |
| Preprotein translocase subunit YajC | *yajC* | -4.3 | - |
| Membrane protein YidC | *yidC* | -3.7 | - |
| Protein translocase subunit SecA | *SecA* | -2.6 | - |
| Protein-export protein SecB | *SecB* | 6.0 | - |
| Protein TatA | *TatA* | -7.4 | -1.5 |
| Signal peptidase I (Spase I) | *lepB* | -5.7 | - |
| Signal recognition particle protein (SPR) | Ftsy | 1.5 | 1.3 |
| Protein-export membrane protein SecF | *secF* | -2.4 | - |
| OMP assembly factor BamA | *bamA* | -2.0 | - |
| OMP assembly factor BamD | *yfiO* | -2.8 | - |
| OMP assembly factor BamE | *smpA* | -3.8 | - |
| OMP assembly factor BamC | *nlpB* | -2.1 | - |
| Chaperone SurA | *surA* | -7.6 | -1.7 |
| Outer membrane protein X | *ompX* | -3.3 | - |
| Outer membrane protein A | *ompA* | -1.9 | - |
| Suppressor of ompF assembly mutants | *asmA* | -3.6 | - |
| ***Two-Component Systems*** | | | |
| Sensor protein PhoQ | *phoQ* | -2.2 | - |
| Phosphate-binding protein PstS | *pstS* | -19.1 | -3.6 |
| Acid phosphatase PhoC | *phoC* | -4.7 | - |
| Two-component sensor protein CpxA | *cpxA* | -2.8 | - |
| Multidrug resistance protein MdtA | *yegM* | -3.0 | - |
| Aerobic respiration control sensor protein ArcB | *arcB* | -2.5 | - |
| Negative response regulator ArcA | *arcA* | 6.4 | - |
| Outer membrane lipoprotein RcsF | *rcsF* | -7.2 | - |
| Cryptic nitrate reductase 2 alpha subunit | *narZ* | -23.2 | - |
| Cryptic nitrate reductase 2 beta subunit | *narY* | -15.7 | - |
| Fumarate reductase flavoprotein subunit | *frdA* | -2.2 | - |
| Succinate dehydrogenase iron-sulfur subunit | *frdB* | -2.5 | - |
| Transcriptional regulatory protein RcsB | *rcsB* | 5.5 | - |
| Zn-binding periplasmic protein | *zraP* | -7.9 | - |
| ATP-binding protein of glutamate/aspartate transport system | *gltL* | -5.9 | -2.5 |
| Glutamate/aspartate transport protein | *gltJ* | -6.9 | - |
| Cyclic AMP receptor protein | *crp* | 5.1 | 1.0 |
| Cytochrome oxidase bd-II | *cydA* | -12.4 | - |
| Protein tyrosine phosphatase | *wzb* | 3.2 | - |
| Pectinesterase | *ybhC* | -3.2 | -1.3 |
| Alkaline phosphatase PhoA | *phoA* | - | -3.6 |
| Putative outer membrane protein SilC | *CusC* | - | -2.3 |
| ***CAMPs resistance*** | | | |
| Undecaprenyl phosphate-alpha-L-Ara4N transferase ArnT | *arnT* | -5.8 | -1.1 |
| Copper homeostasis protein | *cutF* | -7.6 | -1.8 |
| Peptide transport periplasmic protein SapA | *sapA* | -2.5 | - |
| Peptide transport protein SapF | *sapF* | -2.7 | - |
| Two-component sensor protein CpxA | *cpxA* | -2.8 | - |
| Acridine efflux pump | *acrA* | -1.4 | - |
| Putative enzyme YcfS | *ycfS* | -14.4 | - |
| Sensor protein PhoQ | *phoQ* | -2.2 | - |
| N-acetylmuramoyl-L-alanine amidase | *amiA&amiC* | -6.0 | -4.9 |
| Thiol: disulfide interchange protein | *dsbA* | -12.7 | -3.4 |
| Peptidyl-prolyl cis-trans isomerase | *ppiA* | -9.7 | -3.4 |
| ***RNA degradation and nucleotide excision and repair*** | | | |
| Ribonuclease E (RNase E) | *rne* | -1.4 | 1.5 |
| ATP-dependent RNA helicase RhlB | *rhlB* | -1.3 | 1.0 |
| Enolase | *eno* | 7.9 | 1.6 |
| ATP-dependent RNA helicase DeaD | *deaD* | -4.0 | - |
| ATP-dependent 6-phosphofructokinase | *KPN_04193* | 4.7 | - |
| 60 kDa chaperonin (GroEL protein) | *groL* | 6.0 | 2.0 |
| Polyphosphate kinase | *ppk* | -2.3 | 2.8 |
| Poly(A) polymerase I (PAP I) | *pcnB* | -3.5 | - |
| RNA-binding protein Hfq | *hfq* | - | 5.7 |
| UvrABC system protein A (UvrA protein) | *uvrA* | -2.9 | 1.1 |
| DNA helicase | *uvrD* | - | 4.7 |
| ***β-lactam resistance*** | | | |
| bifunctional penicillin-binding protein 1a | *mrcA* | -2.4 | - |
| Periplasmic oligopeptide-binding protein | *Opp* | -13.1 | -2.3 |
| Acridine efflux pump | *acrA* | -1.4 | - |
| TEM Beta-lactamase Class A | *bla* | -4.4 | -2.5 |
| class A extended-spectrum beta-lactamase SHV-12 | *KPN_RS27220* | - | -2.5 |
| ***Quorum sensing*** | | | |
| Autoinducer 2-binding protein LsrB | *yneA* | -10.3 | - |
| 3-hydroxy-5-phosphonooxypentane-2,4-dione thiolase | *yneB* | 8.4 | - |
| Periplasmic oligopeptide-binding protein | *Opp* | -13.1 | -2.3 |
| Membrane protein insertase YidC | *yidC* | -3.7 | - |
| Periplasmic murein tripeptide (L-Ala-gamma-D-Glut-m-DAP) permease | *mppA* | -13.6 | -1.8 |
| Oligopeptide transporter ATP-binding component | *oppD* | -9.1 | - |
| Oligopeptide transport protein | *oppA* | -11.0 | -1.9 |
| Protein translocase subunit SecA | *secA* | -2.6 | - |
| Preprotein translocase subunit YajC | *yajC* | -4.3 | - |
| Protein-export protein SecB | *secB* | 6.0 | - |
| Cyclic AMP receptor protein | *crp* | 5.1 | 1.0 |
| Putative ABC transporter periplasmic binding protein | *ydcS* | -22.3 | -4.2 |
| High-affinity branched-chain amino acid transport protein | *livJ* | -19.0 | -2.4 |
