# Supplementary figures and images for: Comparative Proteomics of Outer Membrane Vesicles from Polymyxin-Susceptible and Extremely Drug-Resistant Klebsiella pneumoniae
Source: mSphere. 2023 Jan 9;8(1):e00537-22. doi: 10.1128/msphere.00537-22 (PMC9942579; doi:10.1128/msphere.00537-22)

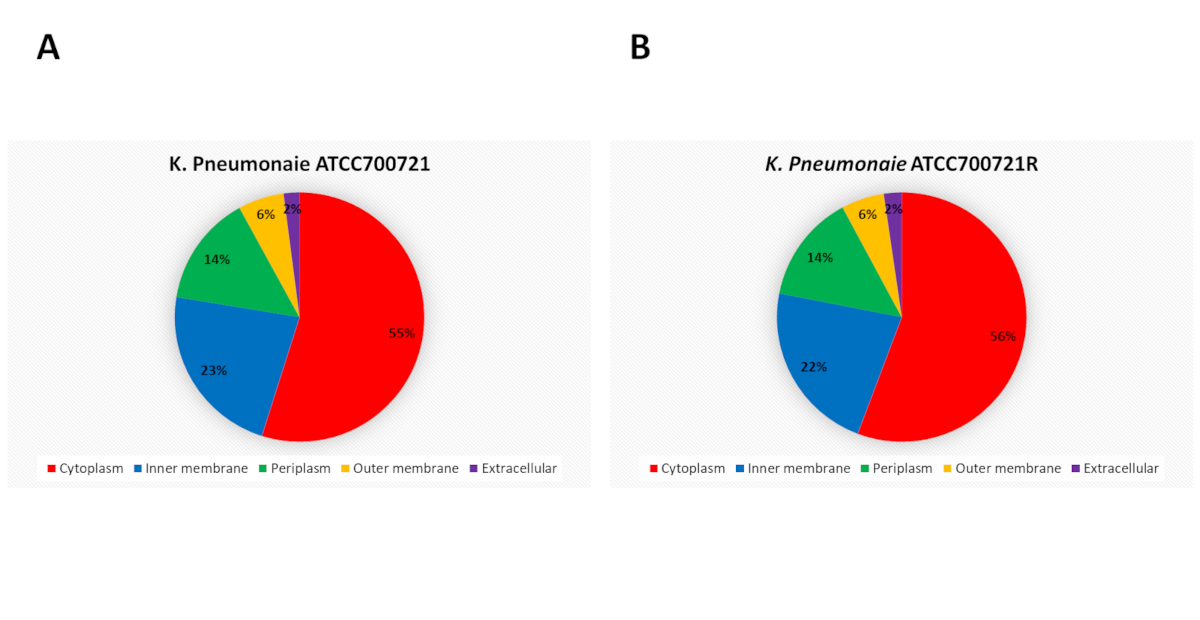

Supplement: FIG S1 [file msphere.00537-22-s0002.tif]

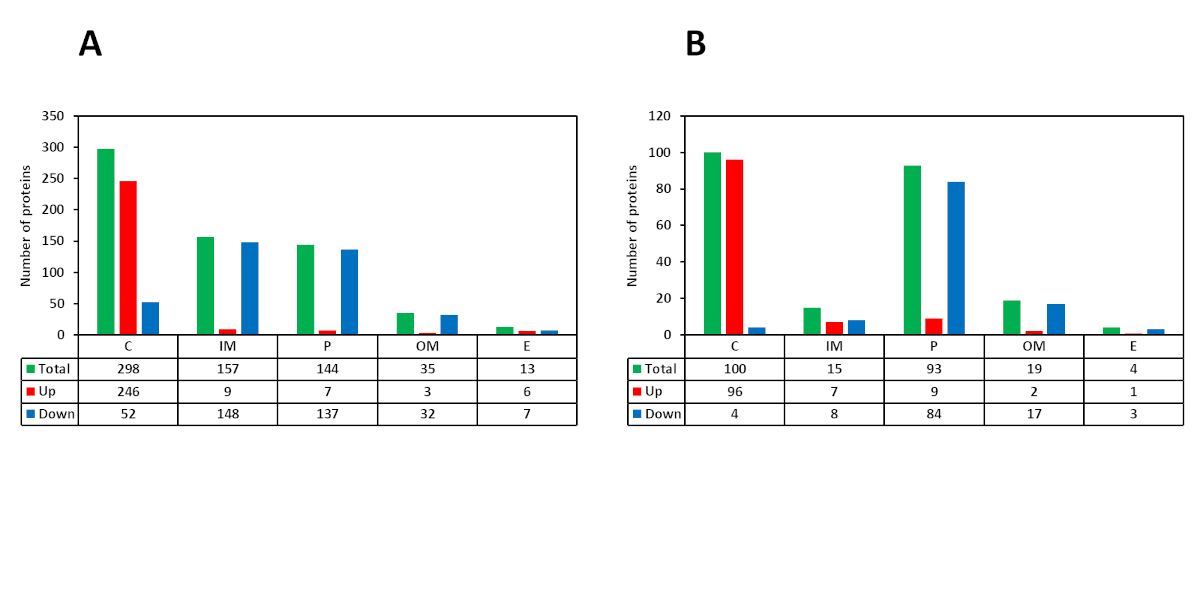

Supplement: FIG S2 [file msphere.00537-22-s0003.tif]

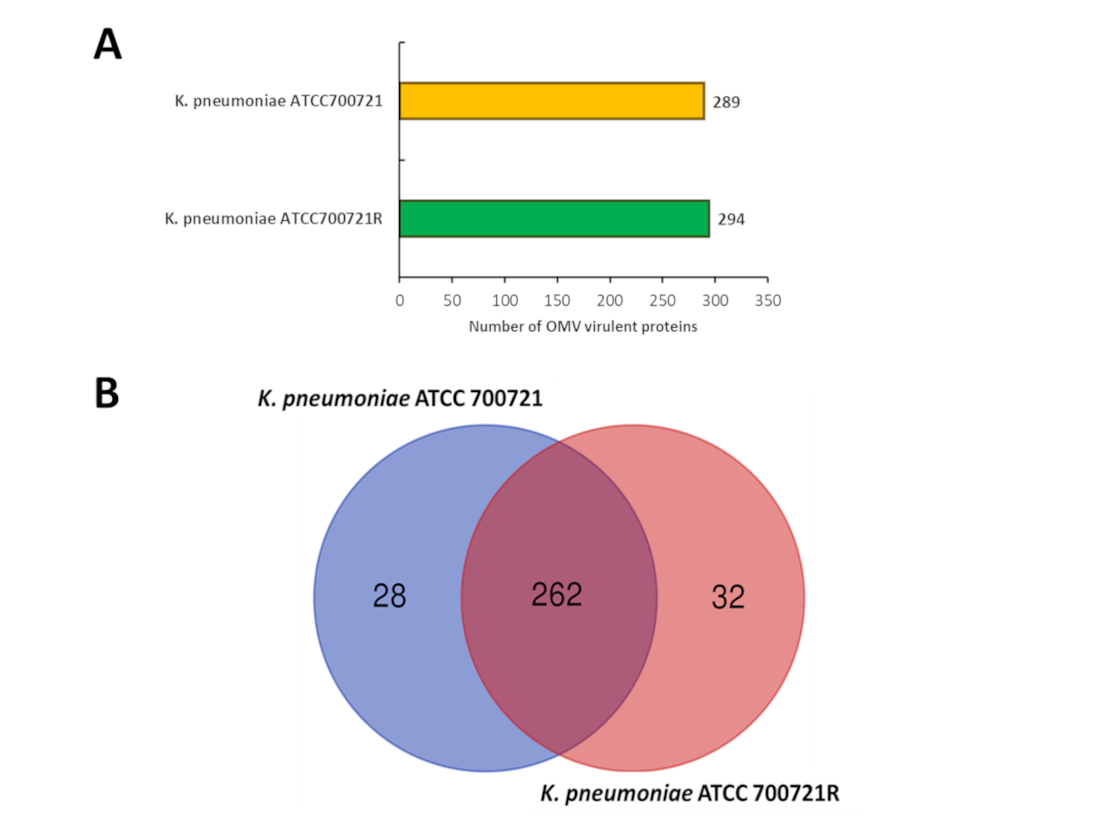

Supplement: FIG S3 [file msphere.00537-22-s0004.tif]
